# Editorial: Endocrine disruptors in gut endocrinology

**DOI:** 10.3389/fendo.2024.1536495

**Published:** 2024-12-13

**Authors:** Velmurugan Ganesan, Marica Colella, Luigi Santacroce

**Affiliations:** ^1^ Chemomicrobiomics Laboratory, Department of Biochemistry & Microbiology, KMCH Research Foundation, Coimbatore, India; ^2^ Department of Theoretical and Applied Sciences (DiSTA), eCampus University, Novedrate, Italy; ^3^ Interdisciplinary Department of Medicine, Section of Microbiology and Virology, School of Medicine, University of Bari, Bari, Italy

**Keywords:** endocrine gut, endocrine disruptors, gut microbiota, gut-brain axis, intestinal dysbiosis, hormonal imbalance

## Introduction

Interactions between the endocrine system, gut microbiota, and endocrine disruptors are multifaceted study areas with significant implications for human health. The Research Topic “*Endocrine Disruptors in Gut Endocrinology*” aims to offer an up-to-date overview of these components and their interconnections.

Disruptions in the endocrine system compromise hormone production, potentially leading to various health issues, including obesity, diabetes, gastrointestinal and reproductive disorders, and endocrine-related cancers.

Conversely, gut microbiota plays several essential roles, including immune and hormonal regulation, influencing systemic inflammation and susceptibility to infections, and helping the digestion of complex carbohydrates, fats, and proteins, producing short-chain fatty acids (SCFAs) and other metabolites that contribute to gut health and systemic metabolism.

Due to the close connection of the gut and oral microbiotas, any intestinal dysbiosis may alter the physiologic state of the oral one, influencing local and systemic conditions (1).

For this reason, gut microbiota may be a useful tool for both diagnosis and therapy in certain clinical conditions (2).

## Endocrine disruptors

Endocrine disruptors are synthetic or natural chemicals that can interfere with the body’s endocrine (hormonal) system. They can mimic or inhibit hormones and influence various pathways, potentially leading to adverse health effects (3).

Common sources of endocrine disruptors include molecules usually present in personal care products as well as in vegetables (i.e., phthalates, parabens, and organophosphates), and plastics (e.g., bisphenol A - BPA) (4).

## Gut microbiota and endocrine disruptors

### Alterations in gut microbiota composition

Exposure to endocrine disruptors can lead to changes in the composition and diversity of gut microbiota, contributing to dysbiosis. Dysbiosis is associated with several disorders, including obesity, metabolic syndrome, acute and chronic renal diseases, and inflammatory bowel diseases.

### Metabolism of endocrine disruptors

Gut microbiota can metabolize endocrine disruptors, potentially detoxifying them or, conversely, converting them into more harmful metabolites. The interplay between gut bacteria and these chemicals influences systemic exposure levels and toxicity.

### Inflammation

Dysbiosis of gut microbiota from endocrine disruptor exposure may contribute to increased gut permeability (or “leaky gut”), which can lead to systemic inflammation and further endocrine system imbalances.

## Gut microbiota and the endocrine system

### Hormonal regulation

Gut microbiota metabolism affects hormone levels, such as the deconjugation of estrogens and their subsequent reabsorption or excretion. This can influence estrogen levels and activity in the body, impacting reproductive health and risks of hormone-mediated cancers.

Furthermore, recent research shows that intestinal microbial metabolites are fundamental for local neuroendocrine regulation and drug metabolism. In detail, such metabolites can allow a higher efficacy of anti-tumor drugs and diabetes control. (Liu et al.; Zhu et al.; 5, 6; Toft et al.).

### Interaction with the gut-brain axis

The gut microbiota can influence the endocrine system through the gut-brain axis, affecting stress responses and behavior. This interaction may affect mental health, especially regarding hormonal influences (7).

## The Research Topic contributions

The Research Topic “*Endocrine Disruptors in Gut Endocrinology*” hosts four published manuscripts reporting original contributions in the field.

Liang et al. offer a notable contribution to personalized medicine with the paper “*Diagnostic model for predicting hyperuricemia based on alterations of the gut microbiome in individuals with different serum uric acid levels*”. They defined the differences in the gut microbiome among participants with different uric acid levels to develop a model to predict hyperuricemia based on 12 new microbial biomarkers.

Liu et al. contributed a real-world vision of commercially available glucagon-like peptide-1 receptor agonists (GLP-1 RAs) efficacy in glycemic control. Their paper “*Association between different GLP-1 receptor agonists and gastrointestinal adverse reactions: A real-world disproportionality study based on FDA adverse event reporting system database*” provides valuable evidence for selecting appropriate GLP-1 RAs to avoid undesired gastrointestinal side effects.

Zhu et al. published the paper “*Examination of the mechanism of Piezo ion channel in 5-HT synthesis in the enterochromaffin cell and its association with gut motility*”. It is an interesting contribution to understand better how human-derived enterochromaffin cells that functionally express Piezo ion channels are associated with cellular mechanosensation via serotonin in peristalsis regulation.

Toft et al. also contributed in understanding intestinal peristalsis. Their paper “*Microbial metabolite p-cresol inhibits gut hormone expression and regulates small intestinal transit in mice*” shows how *in vitro* the microbial metabolite p-cresol can suppress transcript levels of gut hormones and regulate small intestinal transit in mice by interference with glucagon-like peptide-1 (GLP-1) secretion.

## Conclusion

The reciprocal interactions between the endocrine system, gut microbiota, and endocrine disruptors represent a novel and critical area of biomedical research. Understanding how these components interact can provide insights into the pathophysiologic mechanisms underlying various clinical conditions and diseases, and help develop strategies for their prevention and treatment.

Collectively, the studies published in the Research Topic “*Endocrine Disruptors in Gut Endocrinology*” emphasize the complex interplay between the gut microbiome, gastrointestinal physiology, and systemic health, showcasing significant advancements in understanding and managing various conditions. Liang et al. highlighted the predictive power of microbiome alterations in hyperuricemia, offering a step ahead in personalized medicine. Liu et al. provided critical insights into optimizing GLP-1 receptor agonist selection to balance glycemic control and minimize common gastrointestinal adverse effects. Zhu et al. explored mechanosensation via serotonin in gut motility, while Toft et al. demonstrated how microbial metabolites like p-cresol can modulate gut hormone expression and intestinal transit. Together, these findings refine the importance of the gut and its microbiome in health and disease, paving the way for innovative diagnostic tools and therapeutic strategies.

Future research is essential to clarify these relationships and assess the potential for interventions, such as lifestyle modifications and probiotics, to counteract the negative health effects of endocrine disruptors.
